# Meeting Reports

**DOI:** 10.1155/S1463924678000139

**Published:** 1978

**Authors:** Peter B. Stockwell, D. G. Porter


period during which the valve is either on or off and the rate of pumping. An air supply from a cylinder or air line at a pressure of a few kilograms per square centimetre is required.

The device is very flexible, the rate of flow can be quickly adjusted by altering the timer settings or changing the syringe size or stroke and it will work for long periods without failure. It is not suitable for continuous analysis systems due to the pulsating flow but it can be used for transferring any organic or inorganic liquid at regular intervals or rates. The pressure developed can exceed 25 kilograms per square centimetre and will depend on the air line pressure.

The cost of the syringe, timer, valves and cylinder is about £80. Proprietary items were purchased from:—

Syringe — Hamilton, Gas-tight
Timer —F. R. Electronics Ltd., Cycling Timer TS-CT2C
Valve —H. Kuhnke Ltd., NWR spool valve
Cylinder —H. Kuhnke Ltd., Miniature cylinder

REFERENCES
[1] C. J. Jackson, D. G. Porter, A. L. Dennis and P. B. Stockwell (1978) *Analyst,* **103,** 317
[2] R. G. Lidzey, C. J. Jackson and D. G. Porter (1977) *Laboratory Practice,* **26,** 400

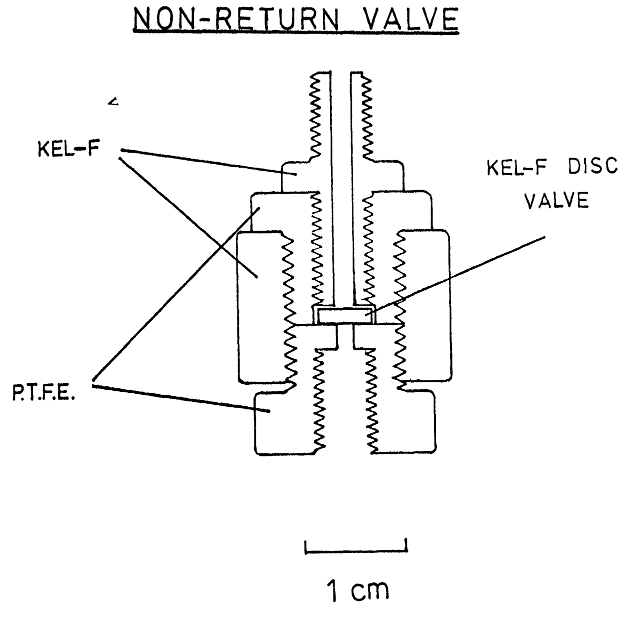

*Figure 1*

### Meeting Reports

### Chromatography Symposium

The 12th International Symposium on Chromatography was held in Baden-Baden, Germany, from the 25th-29th September 1978. The meeting was organised by the Gellschaft Deutscher Chemiker in conjunction with the Chromatography Discussion Group, Groupement pour l'Avancement des Methodes Spectroscopiques et Physiocochimiques d'Analyse, and Arbeitskreis Chromatographie der Fachgruppe Analytische Chemie. It was attended by over 700 delegates from more than 25 countries. The city of Baden-Baden, particularly the conference centre itself, was an ideal setting for such a meeting. As delegates converged on Baden-Baden they were greeted by splendid sunshine which lasted until midway through the meeting when the rain began to dampen one's spirits slightly. The former added to the warmth of the meeting and the latter no doubt helped account for the high attendance at the various scientific sessions. These were organised as a series of plenary, contributed and review lectures and these, like the weather, varied between the good and the not so good. Each lecture had previously been submitted and for the most part fully refereed to appear in a concise conference handbook produced by the Publishers Elsevier and they should be congratulated on this achievement. Such a format does, however, deviate from the original style of these symposia where the papers were available in a pre-printed form prior to the symposia and the handbook itself included any relevant discussion on the papers. This would seem more acceptable and allows speakers to concentrate on the major interest in their papers particularly on recent findings and for more detailed and fruitful discussions. This comment is, however, not meant to detract from the success that the organisers did in fact achieve — the programme catered for a wide range of tastes both with regard to the technique of chromatography and from the areas of applications these ranged from medical applications, and spirit analysis through to such problems as geochemical analysis. The meeting was however highly successful in providing a pleasant meeting place for chromatographers generally to discuss their problems with other workers and instrument companies.

The scientific content of the symposium was varied and interesting and included papers dealing with the examination of materials as diverse as geochemical substances, gin and biological fluids. Capillary column GC and GC-MS figured to a great extent, whilst less sophisticated techniques such as exclusion chromatography also received ample coverage.

Schomburg, as one has come to expect, gave an excellent review of the application of capillary gas chromatography; in this the value of two dimensional chromatographic analysis, or column switching, was indicated. Also with regard to capillary columns, there was a stark contrast between the approach of Guichon with columns over 1 km long and of the rather short columns used by Liberti. Each approach offers a number of possibilities for the analyst.

An excellent review lecture by E. Jellum (Oslo) illustrated the usefulness of capillary column GC-MS in conjunction with a spectral retrieval system, for the analysis of blood and urine samples to enable various metabolic disorders to be pinpointed. Dr. Jellum indicated that analysis was being carried out on blood given by donors over a period of years in an attempt to establish the presence of any "marker" substance that could be correlated with the onset of cancer. The implications of this study are manifest and demonstrate the potential benefits of improved chromatographic technology to the bio-sciences.

Whilst the subject of the lecture programme barely touched on the problems and applications of automation, the exhibition did include some aspects of automation and mention of some of these are made here. It is perhaps disappointing that those aspects of automation which receive most attention here are those related to microprocessor control and data processing and sample injection; the only total automation system due to be presented by Technicon did not in fact appear. It was probably most pleasing to see the increasing importance placed by instrument manufacturers on the provision of column switching techniques which significantly improve the performance specification of many separations. This is particularly true for capillary gas chromatography and both Packard and Siemens have produced viable systems the former in association with a microprocessor-controlled gas chromatograph. Literature on these devices was not currently available but the Siemens instrument, whilst being expensive, and to a slightly lesser extent the Packard system, allow considerable flexibility with regard to heat cutting, column switching, peak trapping and the various other options open to a separation scientist. Pye Unicam exhibited an intelligent microprocessor-controlled column switching device developed at the Phillips Laboratory, Salford, Surrey, specifically designed for HPLC where there has, as yet, been little effort to explore the possibilities of column switching. The device can also be readily applied to gas chromatography.

Several sample introduction devices were shown, of these two from Kipp Analytical and from Waters are relatively recent innovations. In the Kipp Autoinjector the vials are fed under gravity to the sampling needle instead of using a more conventional turntable concept. This, together with the reduction of moving parts results in a high degree of reliability. However it is then impossible to introduce samples into a run at any time other than the start of a sequence or to sample a number of times from one container since the sample is discarded once injected. For routine tasks the approach offers potential cost savings, however the turntable approaches, particularly where a segmented tray is used in conjunction with a microprocessor controller, offers considerable versatility and flexibility. In contrast to the Kipp system the Waters WISP (Waters Intelligent Sample Processor) is a far more complicated device. All injection parameters are independently programmable to achieve the desired sample injection conditions, the microprocessor capability is used to provide simplified controls and self-checking diagnostics. For example, the unit automatically counts and memorises vial positions so that new samples can be added during a run. The WISP can also communicate with other intelligent modules in the Waters range.

The use of headspace analysis often can overcome the need to carry out complicated column separations and in addition to the conventional automatic headspace analyser system Perkin Elmer also exhibited a semi automated system to handle six samples as a batch which can be linked to the Sigma range of instruments.

As could be easily forecast, microprocessors are increasingly seen on instrument companies' stands. Their integration in many instruments was included at this symposium. The Packard instrument has already been briefly mentioned, but soon almost all gas chromatographs, and indeed liquid chromatographs as well, will not be complete without them. However, the predicted cost savings that were said to be concomitant with the introduction of the advanced techniques of electronics are, I fear, a long way off. Most of the instruments using microprocessor technology do little more than emulate the accepted manual approaches with addition of control of the various setting parameters and the addition of data integration and some calculation inbuilt. A little more vision could, in fact, have been more rewarding, the only instrument for example using a visual display monitor was the Varian liquid chromatograph. This uses, in fact, a very small screen where a conventionally sized visual display unit with perhaps a graphics capability would have opened up a whole new range of possibilities. Such approaches will undoubtedly come as users become more aware of the possibilities of integrating microprocessors into instruments and become prepared to pay the cost of instruments which involve such a high development cost.

However, it's fairly clear that symposia of this kind are a very useful manner in which to encourage the verbal interchange between the users from wide fields of interest and the manufacturers. the next symposia is planned to be held in Cannes, France in 1980 and the organisers of this have been set a very high standard by the meeting at Baden-Baden.

*Peter B. Stockwell*

### Safety and Automation

A joint meeting on the above topic organised by the Automatic Methods Group, and the N.W. Region of the Chemical Society, U.K., Analytical Division was held in Chester on 5th October, 1978.

The first part of the meeting was a visit to the Bass Charrington Production Plant at Runcorn. This was exceedingly interesting and often quite surprising, the plant which is one of the most modern in the U.K., whilst obviously very efficient, had no computer on site. The contrast between continuous and batch operations was also highlighted in the tour and the continuous operations seem to have made little impact because of the long time involved in stabilising the yeast column prior to production of the beer.

In the afternoon session Dr. Jackson of the Health and Safety Executive, London, presented an account of the evaluation and subsequent modification of an instrument designed to monitor lead emissions from stacks. The basic design considerations of the instrument, the need to provide these measurements, and the suitability of the instrument, which is based on atomic fluorescence principles, for the task were described. The second talk, given by Dr. Allen, formerly of G. D. Searle, High Wycombe, described an interesting approach to the containment of automatic instruments particularly in a microbiological sense. The developments which had been carried out for the Research Group at Searle have many implications in other areas particularly those in which there is concern about the hazardous nature of the materials handled, for example in the analysis of carcinogens. Problems associated with the electrical safety of instruments in flammable atmospheres were described by R. Moore of ICI Plastics, Welwyn, U.K. in an amusing and enlightening lecture. The legal requirements on an instrument designer were set out and many useful ways of minimising or avoiding the risks of explosions were illustrated. The meeting was very interesting and enjoyable for the participants, and, in spite of a relatively low attendance, was considered by the organisers to have been successful.

*D. G. Porter*

